# Notes

**Published:** 1898-10-15

**Authors:** 


### NOTES.

The Chelsea Hospital for Women, after undergoing its annual cleaning, is now again fully open to patients. Not more than a third of the beds were closed at a time.

The Birmingham Homœopathic Hospital is to be gradually extended. One part of the additional buildings has just been opened, and consists of a small block to be utilised as quarters for the nurses.

A systematic course of lectures and demonstrations for sanitary inspectors has been arranged at King's College. The introductory lecture will be given on Friday, October 14th, at eight o'clock, by Professor W. J. Simpson, M.D. Mr. McKinnon Wood, chairman of the London County Council, will preside.

The receipts of the Hospital Saturday Fund from January last to September amounted to £8,429 17s., a decrease of £1,458 17s. on the corresponding period in the previous year. Hospital Saturday this year will be held on October 15th, and the annual meeting on October 29th.

Dr. Barnardo has opened a Home for Incurable Children at Bradford. It is intended for twenty-five inmates, who will be admitted absolutely free.

A new isolation hospital is about to be erected at Southampton for the use of the borough, and the foundation-stone has now been laid by the Mayor. It provides accommodation for 70 beds, with provision for considerable extension in the future. The wards will be situated in pavilions, of which one is for private patients.

Extensions have recently been made at the Berwick Infirmary, consisting of improvements and additions to the administrative department. The extension serves as a Diamond Jubilee memorial, and a terra-cotta medallion of the Queen has been placed in the entrance lobby of the infirmary, below it being placed a plate recording date of the extension and the event of which it serves as a memorial.

An isolation hospital has been opened at Northleach. It is the gift of Lord Eldon, and has been opened by him. The Rural District provided the site, which is an excellent one. The accommodation consists of two wards, with five beds and two cots in each. The wards are each 30 ft. by 20 ft. Accommodation for the matron is situated between the two wards. The temporary hospital is to be utilised for laundry, mortuary, and disinfecting purposes. There is also a caretaker's cottage.

A new home has been established at Manningham, near Bradford, mainly through the generosity of Mr. and Mrs. Cawthra, of Horton, near Bradford, at a cost of nearly £10,000. The institution, which is to serve as a memorial to their son, is for the reception of cancer patients and incurables. There are beds for 24 inmates, dispersed over five wards. The aim of the donors has been to render the establishment as homelike and comfortable as possible. Lifts are provided, and the best appliances and sanitary arrangements have been aimed at. The furnishing of the wards, which had to be subscribed for by the public, has been partly undertaken by generous friends.

Complaint has recently been made by the Montrose Parish Council in regard to the action of the managers of the Montrose Infirmary in discharging a woman who had been treated for fracture of the thigh at a time when her confinement was nearly due, the woman, in fact, giving birth to a child only a few hours after her dismissal. The charge, which was in reality levelled at the medical staff, was discussed at the last monthly meeting of the Infirmary Board, and in the end a motion was accepted exonerating the staff from blame in the matter. From the account given by Dr. Stone it seems clear that the only reason why she was discharged was the risk she would have had to run if she had remained to be confined in a surgical hospital. As to the quaint suggestion made by some members of the House Committee that she might have been removed to the infectious ward at the back of the hospital, which was not then in use, we need say nothing. The very statement of the suggestion is sufficient to condemn it.

The late Mr. James B. Thomson, whose death occurred recently, has left a fortune of nearly £200,000, the greater portion of which will be divided among the Glasgow charities.

A new home has been opened at the Royal Albert Asylum for Idiots at Lancaster. The building is the gift of Sir Thomas Storey, and is intended to accommodate 40 feeble-minded girls, who will be employed in duties suited to their capacities in the asylum. Lady Bective opened the home on the occasion of the annual meeting of the asylum, and Sir John Hibbert unveiled a bust of Sir Thomas Storey, placed in the new institution on the same occasion. Sir John Hibbert also laid the foundation-stone of quarters for epileptic patients. It is obvious that the Lancaster Asylum will soon be one of the most complete establishments for the treatment of the mentally afflicted in the country.

At the last meeting of the Plymouth Board of Guardians the report of the sub-committee of the Town Council, in regard to the complaints made by the Guardians as to the treatment of certain children sent from the workhouse to the isolation hospital, and paid for by them, received consideration. The Guardians stick to their guns. They maintain that they were only doing their duty to the poor, who are placed in their care, in investigating as fully as they were able the complaints which had been made in regard to their treatment in the isolation hospital; they profess themselves entirely unconvinced by the report of the sub-committee of the Town Council, according to which the charges which had been brought forward were unfounded; and they criticise very severely the mode in which this sub-committee conducted its inquiry. Much as we regret any unpleasantness between different authorities, we cannot but feel that, on the whole, the inhabitants of Plymouth are to be congratulated on the fact that this matter has been given publicity. Little personal asperities will, we hope, be soon forgotten, but even if not it is better so than that such abuses as those which the Guardians still maintain to have taken place should continue to exist.

